# Family and parenting factors are associated with emotion regulation neural function in early adolescent girls with elevated internalizing symptoms

**DOI:** 10.1007/s00787-024-02481-z

**Published:** 2024-06-04

**Authors:** Sylvia C. Lin, Elena Pozzi, Christiane E. Kehoe, Sophie Havighurst, Orli S. Schwartz, Marie B. H. Yap, Junxuan Zhao, Eva H. Telzer, Sarah Whittle

**Affiliations:** 1 Department of Psychiatry, The University of Melbourne, Melbourne, Australia; 2 Orygen, Melbourne, Australia; 3 Centre for Youth Mental Health, The University of Melbourne, Melbourne, Australia; 4 Mindful, Centre for Training and Research in Developmental Health, The University of Melbourne, Melbourne, Australia; 5 Turner Institute of Brain and Mental Health, School of Psychological Sciences, Monash University, Melbourne, Australia; 6 Melbourne School of Population and Global Health, The University of Melbourne, Melbourne, Australia; 7 Department of Psychology and Neuroscience, University of North Carolina at Chapel Hill, North Carolina, USA

**Keywords:** Parenting, Early adolescents, Emotion regulation, fMRI

## Abstract

A prominent tripartite model proposes that parent role modeling of emotion regulation, emotion socialization behaviors, and the emotional climate of the family are important for young people’s emotional development. However, limited research has examined the neural mechanisms at play. Here, we examined the associations between family and parenting factors, the neural correlates of emotional reactivity and regulation, and internalizing symptoms in early adolescent girls. Sixty-four female adolescents aged 10–12 years with elevated internalizing symptoms completed emotional reactivity, implicit (affect labeling) and explicit (cognitive reappraisal) emotion regulation tasks during functional magnetic resonance imaging. Positive family emotional climate was associated with greater activation in the anterior cingulate and middle temporal cortices during emotional reactivity. Maternal emotion regulation difficulties were associated with increased frontal pole and supramarginal gyrus activation during affect labeling, whereas supportive maternal emotion socialization and positive family emotional climate were associated with activation in prefrontal regions, including inferior frontal and superior frontal gyri, respectively, during cognitive reappraisal. No mediating effects of brain function were observed in the associations between family/parenting factors and adolescent symptoms. These findings highlight the role of family and parenting behaviors in adolescent emotion regulation neurobiology, and contribute to prominent models of adolescent emotional development.

**Supplementary Information:**

The online version contains supplementary material available at 10.1007/s00787-024-02481-z.

## Introduction

Early adolescence is a developmental period characterized by a flux of biological and socio-emotional changes, and has been associated with a heightened risk of internalizing symptoms such as depression and anxiety, especially in girls [1]. Internalizing symptoms during this period tend to persist into adulthood and have a long-lasting impact on well-being and functioning [2]. Prevention and intervention efforts have focused on identifying pathways leading to the development of internalizing symptoms in young people, and there is converging evidence to suggest that difficulties in emotion regulation could be a transdiagnostic feature of internalizing symptoms [3]. Given the central role of emotion regulation in internalizing difficulties, it is imperative to understand factors that may influence emotion regulation development.

A large body of research suggests that parents are amongst the most influential environmental factors in shaping emotion regulation development in children, and continue to be important, in early adolescence [1]. A number of family and parenting factors have been included in an overarching theoretical model – the tripartite model of family impact on children’s emotion regulation and adjustment [4]. This model proposes three interconnected processes through which parents can influence their children’s emotion regulation development, and in turn, internalizing outcomes: (1) children’s observation of parents’emotion regulation, (2) parental emotion socialization behaviors, such as parents’ reactions to children’s emotions and discussions about emotions, and (3) emotional climate of the family, reflected in parenting styles, marital relationships, parent-child relationships, and family emotionality. Evidence from meta-analyses has consistently provided support for this model, such that more adaptive parental emotion regulation, supportive emotion socialization behaviors, and more positive family emotional climate are linked with better emotion regulation skills and lower internalizing problems in young people [5–7].

While behavioral studies have established a link between family and parenting factors and emotion regulation in young people, the associations with neural correlates of emotion regulation remains relatively unexplored. Neural networks supporting emotion regulation undergo substantial development and restructuring in early adolescence. During this period, there is differential development of limbic and prefrontal control regions, proposed to contribute to increased risk of emotion dysregulation and internalizing problems [8]. The developmental changes, coupled with heightened neuroplasticity, render early adolescents particularly sensitive to environmental influences such as parenting [9], which present both vulnerabilities and opportunities for emotion regulation development.

Though limited, existing neuroimaging research largely converges with findings from behavioral studies to suggest an association between family and parenting factors and neural circuits implicated in emotion regulation [10, 11]. Neurobiological models suggest three key processes involved in emotion regulation: emotional reactivity, implicit and explicit emotion regulation. In regards to emotional reactivity, parenting behaviors have been found to associate with child and adolescent brain function in the prefrontal cortex (PFC) and amygdala. In particular, negative parenting behaviors, such as poor monitoring and harsh behavior, have been associated with lower activity in PFC regions, including the ventrolateral prefrontal cortex (vlPFC) [12] and dorsal anterior cingulate cortex (ACC) [13], during emotion reactivity tasks (e.g., passive viewing of emotional stimuli). Findings regarding the associations between parenting behaviors and amygdala activation have been mixed and inconsistent [14–16].

Functional neuroimaging research investigating implicit and explicit emotion regulation has been more limited. Implicit (automatic) emotion regulation involves incidental regulation processes that occur without conscious awareness, and can be captured in neuroimaging paradigms such as affect labeling [17]. Explicit (conscious) emotion regulation refers to effortful processes employed to regulate emotion, and is commonly assessed using paradigms that instruct participants to downregulate their emotional responses to emotional stimuli via strategies such as cognitive reappraisal [17]. While there are studies examining the relationship between parenting and adolescent brain activity during tasks that may engage emotion regulation neurocircuitry (e.g., parental criticism [18], peer evaluation [19] tasks), only two studies have used an emotion regulation paradigm. Findings from these two studies are mixed: Cosgrove et al. (2020) [20] found that adolescents aged 14 to 16 who reported more unsupportive parental emotion socialization showed increased activation in the supplementary motor area and decreased activation in the amygdala and paracentral gyrus during implicit emotion regulation, although they did not observe significant associations during cognitive reappraisal. On the other hand, Telzer et al. (2014) [21] found no association between parental warmth and brain activity during affect labeling in older adolescents with a mean age of 18 years. These findings are difficult to reconcile, given variation in the parenting factors and adolescent age ranges. In addition, despite the critical developmental period of early adolescence, task-based fMRI studies investigating the relationship between family and parenting factors and early adolescent brain function are still lacking, particularly in adolescents with elevated internalizing symptoms. Such investigation would shed light on the role of parenting and family factors in the neurobiological mechanisms underlying emotional reactivity and regulation in adolescents at risk of poor mental health outcomes, and aid the development and tailoring of targeted interventions.

Guided by the tripartite model [4], this study aimed to comprehensively examine the associations between family and parenting factors proposed to influence child emotion regulation and the neural correlates of emotional reactivity and regulation in early adolescents with elevated internalizing symptoms. A secondary exploratory aim of this study was to examine if brain function mediates the relationship between family and parenting factors and adolescent internalizing symptoms. Of note, this study focuses on female adolescents and their mothers given (1) sex differences in the neural correlates of emotional reactivity and regulation [22]; (2) higher rates of internalizing symptoms in females than males during adolescence [1]; (3) maternal influence on adolescent internalizing outcomes has been found to be stronger among females compared to males [23].

In line with prior research, we hypothesized that maternal emotion regulation, maternal emotion socialization behaviors, and the emotional climate of the family would be associated with activation in the PFC and amygdala during emotional reactivity and regulation. Exploratory whole-brain analyses were conducted to investigate other potential significant effects. Given the limited evidence and the lack of clarity regarding the relationship between parenting and different neural processes of emotion regulation (reactivity, implicit, explicit), we did not have specific predictions on the direction of the effects for each process. In addition, based on meta-analytical findings which indicate that child emotion regulation mediates the relationship between family factors and child/adolescent internalizing symptoms [7], we hypothesized that brain function during emotional reactivity and regulation would also mediate this relationship.

## Methods

### Participants

Sixty-four mother-daughter dyads (adolescents age *M* = 11.45 years, *SD* = 0.77, 10–12 years) participated in the study. Female adolescent participants were included if they had elevated internalizing symptoms as determined by scores above the 50th percentile (raw scores^1^ > 44) on the self-reported Revised Children’s Anxiety and Depression Scale (RCADS) [24] at a screening assessment (raw score *M* = 66.84, *SD* = 17.15, Range = 45–115). Given emotion regulation difficulties are implicated in a range of internalizing symptoms, we examined broad internalizing symptoms rather than depression and anxiety symptoms separately. Exclusion criteria included: (1) current diagnosis of a developmental or intellectual disorder as reported by mothers; (2) current use of psychotropic medication; (3) any contraindications to MRI; (4) indications of claustrophobia; (5) history of head trauma or loss of consciousness for 5 min or more; (6) obesity (BMI > 30). Forty-six adolescents were reported by their mothers to be White/Caucasian (71.9%), followed by Mixed Heritage (14.1%) and Asian (7.8%). All participants provided verbal and written consent and were reimbursed $60 AUD for their time for participation. The study was approved by The Royal Children’s Hospital Human Research Ethics Committee (HREC 77,884). Participant demographics are presented in Table S1.

### Measures

All measures have demonstrated good validity and reliability [24–27]. All Cronbach’s alphas (α) below are based on the current study sample.

#### Adolescent internalizing symptoms

Adolescent internalizing symptoms were assessed by adolescent report on the RCADS ( [24], 0 = *Never* to 3 = *Always*). The RCADS includes 47 items and six subscales: social phobia, panic disorder, major depression, separation anxiety, generalized anxiety, and obsessive-compulsive symptoms. Example items include “I feel sad and empty” and “I worry about making mistakes”. A total raw score was used, with higher scores indicating greater internalizing symptoms (α = 0.91).

#### Maternal emotion regulation

Maternal emotion regulation was assessed by mothers’ self-report on the Difficulties in Emotion Regulation Scale (DERS [25], 1 = *Almost never* to 5 = *Almost always*). The DERS includes 36 items comprising six subscales: lack of emotional awareness, lack of emotional clarity, difficulties controlling impulsive behaviors, difficulties engaging in goal-directed behavior, non-acceptance of negative emotional responses, and limited access to effective emotion regulation strategies. Example items include “When I’m upset, I become out of control” and “I have no idea how I am feeling”. A total score was used, with higher scores indicating greater difficulties (α = 0.8).

#### Maternal emotion socialization behaviors

Maternal emotion socialization behaviors were assessed by adolescent report on the Emotions as a Child (EAC [26]) scale (1 = *Never* to 5 = *Very often*). The EAC scale includes 45 items, measuring maternal emotion socialization of sadness, anger, and fear across five domains: reward, punish, override, neglect, and magnify. Six items were reverse scored. Items were summed into supportive (reward subscale, 3 items, α = 0.93) and unsupportive (neglect and punish subscales, 6 items, α = 0.89) emotion socialization subscales based on a previous study^30^. Example items include “When my child was sad, I comforted them” (reward), “When my child was sad, I did not pay attention to their sadness (neglect)”, and “When my child was sad, I told my child to stop being sad” (punish).

#### Emotional climate of the family

Emotional climate of the family was assessed by adolescent report on two subscales from the Parenting to Reduce Adolescent Depression and Anxiety Scale (PRADAS [27], 0 = *Never* to 3 = *Often*). The parent-child relationship subscale (6 items) assesses parental warmth, aversiveness, affection, and emotional availability. The home environment subscale (6 items) assesses family conflict, parental criticism, and parental modeling of conflict management. Example items include “My mom cares about my opinions” and “I hear my parents arguing with each other”. A total score of items across the two subscales was used, with higher scores indicating more positive family emotional climate (α = 0.79).

### fMRI tasks

#### Affect labeling task

**Fig. 1:**
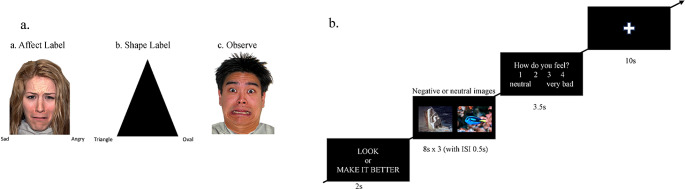
Affect labeling and cognitive reappraisal fMRI tasks

We used a block-design affect labeling task to assess implicit emotion regulation [21]. During this task, participants were presented with negative (sad, fear, angry) faces from the NimStim set (available at http://www.macbrain.org) or shapes. In the ‘affect label’ condition, participants were instructed to label the target emotional face with one of the two emotional word labels below the face using a button box (Fig. 1a). In the ‘observe’ condition, participants were instructed to view the face and press their thumb against the button box to control for the confounding effect of motor activity [28]. In the ‘shape label’ condition, participants were asked to label the target shape with one of the two shape labels [21]. The ‘affect label’, ‘observe’, and ‘shape label’ conditions were presented randomly in a total of 10 blocks, with four blocks each for ‘affect label’ and ‘observe’, and two blocks for ‘shape label’. Each block included six trials, with a fixation of 12s in between blocks. A trial consisted of a face or shape stimulus presented for 4s, followed by a jittered fixation of 1.52–3.03 s. Faces were balanced for gender and race (two-thirds Caucasian and one-third Asian to reflect ethnic representation in Australia). Emotional labels included afraid, angry, sad, miserable, mad, and scared; and shape labels included triangle, rectangle, and oval.

#### Cognitive reappraisal task

We used a block-design cognitive reappraisal task to assess emotional reactivity and explicit emotion regulation [29]. Participants were presented with neutral or negative pictures and were asked to either regulate or observe them naturally. In the ‘reappraisal’ condition, participants were presented with negative pictures and instructed to think about the picture in a way that made them feel better about it. In the ‘look’ condition, participants were instructed to look at the picture and let themselves feel whatever the picture made them feel. There were 12 counterbalanced blocks and three conditions (reappraisal, look negative, look neutral). Each block began with a cue word ‘make it better’ or ‘look’ for 2s, followed by three picture stimuli each presented for 8s. Participants then rated how they were feeling on a scale from 1 (neutral) to 4 (very bad) using a button box (note that ratings were not included in the modeling of these conditions). The rating question was presented for 3.5s and there was an interstimulus interval of 0.5s between each picture stimulus and the rating (Fig. 1b).

All participants completed a practice before the task where they were given examples of reappraisal and were asked to report their reappraisals aloud. The pictures were comparable to previous studies using a cognitive reappraisal task in children and adolescents [29]. To reduce the risk of distress due to exposure to negative images, parents viewed all pictures and notified the research team if they wanted to swap out specific pictures. Two parents requested to replace pictures depicting violence with pictures of similar valence and arousal ratings. Picture stimulus sets and normative ratings are presented in Supplementary Table S2.

### fMRI acquisition and processing

#### fMRI acquisition

Neuroimaging data were acquired on a 3T Siemens TIM Trio scanner with a 32-channel head coil at the Royal Children’s Hospital in Melbourne, Australia. Before the scan, participants were familiarized with the MRI in a mock scanner.

Structural T1-weighted images were acquired as follows: MPRAGE, slice thickness = 0.9 mm, repetition time = 2500ms, echo time = 1.72/3.45/5.18/6.91ms, flip angle = 8◦, field of view = 256 × 240 × 188 mm, matrix size = 284 × 266 × 208 mm, isotropic voxel size = 0.9 mm. The total sequence was 7.6 min. Functional images included T2*-weighted echoplanar images with the following parameters: 60 slices, slice thickness = 2.5 mm, repetition time = 1250ms, echo time = 30ms, flip angle = 90◦, field of view = 255 × 255 × 150 mm, isotropic voxel size = 2.5 mm. The total sequence was approximately 8 min for each task.

#### fMRI preprocessing and first-level analysis

Preprocessing was conducted using the ENIGMA Harmonized Analysis of Functional MRI pipeline (HALFpipe [30], https://github.com/HALFpipe). It performs preprocessing using fMRIPrep, which includes the following default settings: grand mean scaling with a mean of 10,000, spatial smoothing FWMH of 6 mm, ICA-AROMA denoising, temporal gaussian-weighted filter of 125s.

First-level analyses were performed in HALFpipe. Using a general linear model (GLM), predictors for each task condition were convolved with a double-gamma hemodynamic response function. The contrasts of interest were, for the affect labeling task, affect label > observe (implicit emotion regulation), and for the cognitive reappraisal task, look negative > look neutral (emotional reactivity) and reappraisal > look negative (explicit emotion regulation). Results of the affect label > shape label contrast are reported in Supplementary Table S3.

#### Regions-of-interest (ROIs) extraction

Two ROI masks were generated based on the Automated Anatomical Labeling (AAL) atlas [31] using the WFU PickAtlas Tool (version 3.0.5). The PFC ROI mask included precentral gyrus, superior frontal gyrus (SFG), middle frontal gyrus (MFG), and inferior frontal gyrus (IFG) orbital, IFG pars orbitalis, IFG triangular, gyrus rectus, and ACC. Because existing evidence on parenting and adolescent neural function during emotion regulation tasks are limited and inconsistent in terms of which specific PFC region is implicated, we utilized a large PFC mask that allowed us to investigate the specificity of PFC involvement in an exploratory fashion. The amygdala ROI mask included left and right amygdala as we had no a priori hypothesis on the differences between left and right amygdala.

#### Quality control

All preprocessed anatomical images were visually inspected for asymmetry, signal distortion or drop-out and artifacts. Participants with a mean framewise displacement > 0.5 mm were excluded [32] from analyses (*N* = 1 in the affect labeling task, *N* = 2 in the cognitive reappraisal task).

### Statistical analysis

Second-level analyses were conducted using SPM12 implemented in MATLAB R2018b (Mathworks Inc). Separate GLMs were run to test the associations between each of the family and parenting factors and adolescent brain function for each of the task contrasts. Adolescent age was included as a covariate. We did not include race and family income as covariates given the limited variation in our sample. Both hypothesis-driven ROI (PFC, amygdala) and exploratory whole-brain analyses were performed. As such, 4 (parent/family predictors) x 3 (contrasts) x 3 (2 x ROI, whole-brain) analyses were performed. A small volume correction was applied for ROI analyses. For both ROI and whole-brain analyses, an uncorrected voxelwise correction of *p* < .001, and a cluster-level family-wise error (FWE) threshold of *p* < .0125 was applied. This threshold was applied to correct for multiple (i.e., 4) predictors (i.e., 0.05/4 based on a Bonferroni adjustment). Note that we did not additionally correct for the total number of contrasts given such a Bonferroni adjustment may be overly conservative [33].

Whole-brain mediation analyses were conducted using the CANlabMediation Toolbox (https://github.com/canlab/MediationToolbox). Bias-corrected bootstrapping with 1,000 resamples was conducted to test indirect effects of brain function in the relationship between family and parenting factors and adolescent internalizing symptoms. An FDR-corrected threshold of *q* < 0.05 was applied.

## Results

### Behavioral results

#### Bivariate correlations

Correlations between family/parenting factors and adolescent internalizing symptoms are presented in Table 1.

**Table 1:** Correlations between family/parenting factors and adolescent internalizing symptoms

	Mean	SD	1	2	3	4	5
1. Adolescent internalizing symptoms	61.55	17.84	-				
2. Maternal emotion regulation difficulties	82.02	23.27	0.03	-			
3. Supportive emotion socialization	35.23	7.85	-0.18	-0.13	-		
4. Unsupportive emotion socialization	35.47	10.98	0.30*	0.29*	-0.72**	-	
5. Family emotional climate	26.38	5.62	-0.17	-0.23	0.71*	-0.72**	-

*p < .05, **p < .01

### Cognitive reappraisal success

One-way ANOVA showed a significant effect of condition on emotion ratings, *F* (2, 189) = 23.01, *p* < .001, η^2^ = 0.20. Post-hoc tests showed that ratings on reappraisal blocks were significantly lower than look negative blocks, *p* = .020, *d* = 0.36. Ratings on look neutral blocks were significantly lower than reappraisal blocks, *p* < .001, *d* = 0.54, and look negative blocks, *p* < .001, *d* = 0.90. Descriptive statistics of emotion ratings are presented in Fig. S1.

### fMRI results

Results of main task effects are presented in Table S4.

#### Family/parenting factors and neural correlates of emotional reactivity

**Fig. 2:**
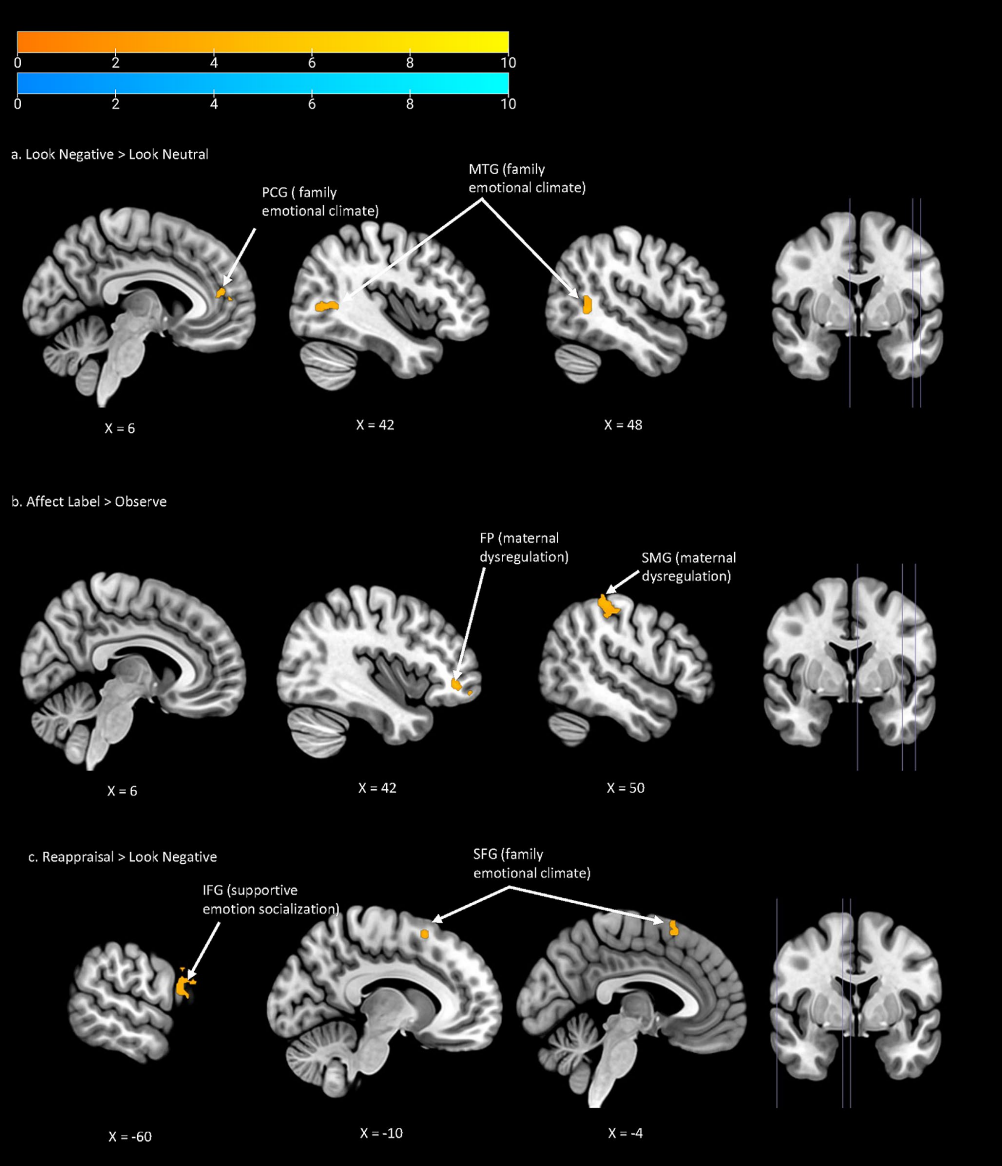
Family/parenting factors and neural correlates of emotional reactivity and regulation in the contrast of (**a**) look negative > look neutral, (**b**) affect label > observe, (**c**) reappraisal > look negative. For all analyses, a cluster-wise FWE threshold of *p* <. 0125 with a voxelwise uncorrected correction of *p* < .001 was applied. FP = frontal pole, IFG = inferior frontal gyrus, MTG = middle temporal gyrus, PCG = paracingulate gyrus, SFG = superior frontal gyrus, SMG = supramarginal gyrus

In ROI analyses of the look negative > look neutral contrast, positive emotional climate of the family was associated with increased activation in the paracingulate gyrus, and was additionally associated with increased middle temporal gyrus activation in whole-brain analyses (Fig. 2a; Table 2).

**Table 2:** Results on the significant associations between brain activation and family/parenting factors controlling for adolescent age

Contrast	ROI or WB analysis	Anatomical region of peak voxel	Family/parenting factor	MNI coordinates			Cluster size	pFWE
				x	y	z		
Look Negative > Look Neutral	PFC ROI and WB	Paracingulate gyrus	Family emotional climate	-4	46	26	80	< 0.001
	WB	Middle temporal gyrus	Family emotional climate	46	-52	2	110	< 0.001
Affect Label > Observe	PFC ROI and WB	Frontal pole	Maternal emotion regulation difficulties	40	44	-4	106	< 0.001
	WB	Supramarginal gyrus	Maternal emotion regulation difficulties	52	-32	58	178	< 0.001
Reappraisal > Look Negative	PFC ROI	Inferior frontal gyrus	Supportive emotion socialization	-60	18	26	61	0.002
	WB	Superior frontal gyrus	Family emotional climate	-12	14	62	88	0.001

FWE = family wise error, PFC = prefrontal cortex, ROI = region of interest, WB = whole brain. Anatomical region of peak voxel was determined based on the Harvard-Oxford Atlas

#### Family/parenting factors and neural correlates of implicit emotion regulation

In ROI analyses of the affect label > observe contrast, maternal emotion regulation difficulties were associated with increased activation in the frontal pole, and was additionally associated with increased activation in the supramarginal gyrus in whole-brain analyses (Fig. 2b; Table 2).

#### Family/parenting factors and neural correlates of explicit emotion regulation

In ROI analyses of the reappraisal > look negative contrast, supportive emotion socialization was associated with increased inferior frontal gyrus activation, and positive emotional climate of the family was associated with increased superior frontal gyrus activation in whole-brain analyses (Fig. 2c; Table 2).

#### Whole-brain mediation analyses

Mediation analyses showed no significant indirect effects of family and parenting factors on adolescent internalizing symptoms via brain function during emotional reactivity and regulation. The associations between internalizing symptoms and brain function are presented in Table S5.

## Discussion

The current study examined the associations between family and parenting factors and neural correlates of emotional reactivity and regulation in a sample of early adolescent girls with elevated internalizing symptoms. Partially consistent with hypotheses, the results showed that maternal emotion regulation, maternal emotion socialization behaviors, and family emotional climate were associated with neural activity in prefrontal, parietal, and temporal regions during emotion regulation processes. However, contrary to hypotheses, we did not find significant associations with amygdala activity, and no mediation effects of brain function in the associations between family and parenting factors and adolescent internalizing symptoms.

We observed an association between positive family emotional climate and increased activity in the paracingulate gyrus/ACC and middle temporal gyrus during emotional reactivity. The ACC, with its bidirectional connections to cortical and subcortical regions, plays a vital role in integrating contextual, affective, and cognitive information to facilitate emotion-related learning [34]. Abnormal functional connectivity between rostral ACC, the amygdala, and prefrontal regions has also been highlighted in adolescent depression [34]. Further, the middle temporal gyrus is implicated in evaluating emotional salience of emotional stimuli and executing regulation initiated by prefrontal regions [35]. Given the functions of the middle temporal gyrus in high-order cognitive processes supporting emotion regulation, the observed pattern of activation may signify the role of positive parent-child relationships and home environments in adolescents’ ability to engage ACC and temporal regions to support emotion processing.

Maternal emotion regulation difficulties were found to be associated with increased frontal pole/vlPFC and supramarginal gyrus activation during implicit emotion regulation. This finding is consistent with research on other adverse social environments, for example, early life stress, which has been found to associate with increased vlPFC activation during affect labeling in early adolescent girls [36]. Given the role of vlPFC/frontal pole in inhibitory control and response monitoring [37], one possible interpretation of the observed frontal activation is that when mothers model maladaptive emotion regulation, adolescents may need to exert greater top-down control effort to regulate emotion subconsciously. The supramarginal gyrus is thought to be involved in semantic working memory [38], likely facilitating the generation of emotional labels during affect labeling. A previous meta-analysis found that patients with depression and anxiety disorders showed increased supramarginal activation during emotion regulation, possibly indicating a compensatory mechanism for less efficient recruitment of other cortical emotion regulation regions [39]. As such, our findings could indicate that adolescents whose mothers reported greater emotion regulation difficulties may exhibit this compensatory activation pattern in the supramarginal gyrus. Alternatively, heightened vlPFC and supramarginal gyrus activity may reflect an adaptive response when maternal scaffolding of emotion regulation development is less accessible [40]. Further research is needed to test these hypotheses.

Supportive maternal emotion socialization behaviors and more positive emotional family climate were associated with increased IFG/vlPFC and SFG/dorsomedial PFC (dmPFC) activity during explicit emotion regulation, respectively. In the context of reappraisal, the vlPFC is implicated in response selection whereas the dmPFC is important for self-reflective processes [37]. The latter observation may suggest that a positive family environment facilitates dmPFC engagement, allowing adolescents to better evaluate and reflect on negative stimuli. Our finding of increased vlPFC activation being associated with supportive emotion socialization, however, is contrary to that of Cosgrove et al. (2020) [20], who used a similar reappraisal task but found no such association in adolescents aged 14 to 16. The inconsistency may be attributed to a smaller sample size and an older participant age group in Cosgrove et al.’s study. Existing research has suggested that early adolescents primarily rely on their parents for emotional support and regulation, while older adolescents increasingly seek these resources from their peers [41]. Taken together, these findings may suggest that supportive maternal emotion socialization behaviors could facilitate vlPFC engagement, supporting early adolescent girls to select goal-directed responses to reinterpret the meaning of negative stimuli. Notably, the effect of maternal emotion socialization was specific to supportive (but not unsupportive) behaviors. This observation is unexpected given evidence linking unsupportive emotion socialization to behavioral measures of poor emotion regulation and mental health [7]. Whether brain function underpinning explicit emotion regulation is particularly sensitive to supportive maternal emotion socialization practices requires further investigation.

Despite the amygdala being commonly implicated in emotional reactivity and regulation, we did not find significant associations between family and parenting factors and amygdala activation. Existing evidence on the effect of parenting on amygdala activity in adolescents has yielded mixed results, with some finding a positive association between supportive maternal parenting behavior and increased amygdala activation to emotional faces [28], and others finding the opposite [14]. One possible explanation is that parenting effects on amygdala activity may be sensitive to emotion types (fear vs. anger vs. disgust) [36]. Our study examined negative emotion more broadly. Thus, more research with greater specificity is needed to elucidate this relationship. In addition, given the suggested importance of bidirectional connections between the amygdala and PFC for emotion regulation [42], it is possible that measures of amygdala connectivity may be more likely to yield associations with family/parenting factors. Future research investigating PFC-amygdala functional connectivity is necessary to better understand this dynamic relationship.

Findings from this study offer theoretical and clinical insights. In particular, maternal emotion regulation was uniquely associated to neural correlates of implicit emotion regulation, whereas supportive maternal emotion socialization and positive family emotional climate were associated with neural correlates of explicit emotion regulation in adolescent girls. It is possible that adolescents subconsciously observe and model how their mothers regulate emotions, thereby influencing neural function during spontaneous and incidental emotion regulation. The effects related to reappraisal may suggest that a positive home environment, where mothers validate adolescents’ emotions and engage in problem-solving, is particularly crucial for nurturing adolescents’ ability to employ cognitive strategies to reinterpret negative events. From a neurodevelopmental perspective, brain networks subserving cognitive reappraisal undergo substantial development throughout adolescence [9]. This developmental plasticity provides a window for intervention. Specifically, targeting family emotional climate and supportive parental emotion socialization practices could scaffold the development of top-down emotion regulation strategies. However, the clinical implications of the study should be considered with caution, given non-significant mediation effects of brain activation in the relationship between family and parenting factors and adolescent internalizing symptoms. Whether greater or less PFC activation during emotional reactivity and regulation is more optimal for internalizing outcomes remains unknown. Important to note, our sample included adolescent girls with elevated internalizing symptoms but generally not meeting the clinical threshold. The observed effects in our study, may be more or less prominent in a clinical sample. Further, the cross-sectional design of the study also limits our ability to establish causality. Longitudinal studies with multiple timepoints and larger sample sized are needed to investigate whether family and parenting factors shape the neurodevelopment of emotion regulation, which in turn, confer risk and resilience to internalizing symptoms in young people.

In addition to a cross-sectional design, the current study has several limitations. First, one predictor variable, maternal modeling of emotion regulation, was assessed by mother-reported difficulties in emotion regulation. While this measure captures self-reported maternal emotion regulation, it does not necessarily capture adolescents’ observation of their mothers’ emotion regulation. Currently, however, there is no validated measure specifically assessing parental modeling of emotion regulation in a home context. Second, we focused on aspects of maternal emotion regulation and parenting. Previous studies have shown that both maternal and paternal parenting, as well as their interactions, play a role in children’s emotion regulation development and internalizing outcomes [43]. It is also important to examine other combinations of parent-child dyads (e.g., fathers and sons). Third, this study did not examine other variables that may additionally influence adolescent emotion regulation neural function, or explain the relationship between the parent and family factors assessed and adolescent emotion regulation neural function. For example, parent internalizing problems may partially explain the association between parent emotion regulation and adolescent emotion regulation neural function. Future work could explore the role this, and other parent/adolescent characteristics and cultural contexts may have. Lastly, participants in the current study were predominantly Caucasians from middle-to-high-income households. The lack of diversity mirrors a broader issue in developmental neuroscience research. Future research should aim to include participants from diverse racial/ethnic backgrounds and various socioeconomic statuses to enhance the generalizability of the findings to a broader community.

In conclusion, this study is the first to investigate the associations between parenting and family factors and neural correlates of emotional reactivity and regulation in early adolescent girls with elevated internalizing symptoms. Our findings underscore the significant impact of maternal emotion regulation, supportive maternal emotion socialization behaviors, and the emotional climate of the family on neural underpinnings of emotion regulation. Importantly, with the increased risk of internalizing symptoms in early adolescents, particularly among girls, these findings may inform family-focused interventions to promote healthy brain function and emotional well-being in young people.

## Electronic supplementary material

Below is the link to the electronic supplementary material.

Supplementary Material 1

## Data Availability

No datasets were generated or analysed during the current study.

## Footnotes

1 RCADS raw scores of > 83 indicate scores above the clinical threshold.
