# Correction: Family and parenting factors are associated with emotion regulation neural function in early adolescent girls with elevated internalizing symptoms

**DOI:** 10.1007/s00787-025-02692-y

**Published:** 2025-04-01

**Authors:** Sylvia C. Lin, Elena Pozzi, Christiane E. Kehoe, Sophie Havighurst, Orli S. Schwartz, Marie B. H. Yap, Junxuan Zhao, Eva H. Telzer, Sarah Whittle

**Affiliations:** 1 Department of Psychiatry, The University of Melbourne, Melbourne, Australia; 2 Orygen, Melbourne, Australia; 3 Centre for Youth Mental Health, The University of Melbourne, Melbourne, Australia; 4 Mindful, Centre for Training and Research in Developmental Health, The University of Melbourne, Melbourne, Australia; 5 Turner Institute of Brain and Mental Health, School of Psychological Sciences, Monash University, Melbourne, Australia; 6 Melbourne School of Population and Global Health, The University of Melbourne, Melbourne, Australia; 7 Department of Psychology and Neuroscience, University of North Carolina at Chapel Hill, North Carolina, USA

**Correction: European Child & Adolescent Psychiatry (2024) 33:4381–4391**

10.1007/s00787-024-02481-z

In the original version of this article, references 21 and 28 were interchanged in the text citation as well as in the reference section. In the text reference citation, reference 21 should be 28 and reference 28 should be 21. In the Introduction section, in the sixth sentence of the fifth paragraph which previously read On the other hand, Telzer et al. (2014) [21] found no association between parental warmth and brain activity during affect labeling in older adolescents with a mean age of 18 years. These findings are difficult to reconcile, given variation in the parenting factors and adolescent age ranges and should have read On the other hand, Chen et al. (2020) [21] found that high levels of supportive maternal emotion socialization at 33 months were associated with greater amygdala activity during affect labeling of angry faces, whereas low levels of supportive maternal emotion socialization were associated with greater amygdala activity during affect labeling of happy faces in adolescents aged 12 to 15. These findings are difficult to reconcile, given variation in the fMRI task paradigms and study designs. In the reference section, ref. 21 which previously read ‘Telzer EH, Qu Y, Goldenberg D, Fuligni AJ, Galván A, Lieberman MD (2014) Adolescents’ emotional competence is associated with parents’ neural sensitivity to emotions. Front Hum Neurosci 8:558’ should read as ‘Chen X, McCormick EM, Ravindran N, McElwain NL, Telzer EH (2020) Maternal emotion socialization in early childhood predicts adolescents’ amygdala-vmPFC functional connectivity to emotion faces. Dev Psychol 56(3):503–515’.

Reference 28, which previously read ‘Chen X, McCormick EM, Ravindran N, McElwain NL, Telzer EH (2020) Maternal emotion socialization in early childhood predicts adolescents’ amygdala-vmPFC functional connectivity to emotion faces. Dev Psychol 56(3):503–515’ should read as ‘Telzer EH, Qu Y, Goldenberg D, Fuligni AJ, Galván A, Lieberman MD (2014) Adolescents’ emotional competence is associated With parents’ neural sensitivity to emotions. Front Hum Neurosci 8:558’.

