# Supplementary material for: Family and parenting factors are associated with emotion regulation neural function in early adolescent girls with elevated internalizing symptoms
Source: Eur Child Adolesc Psychiatry. 2024 Jun 4;33(12):4381–91. doi: 10.1007/s00787-024-02481-z (PMC11618192; doi:10.1007/s00787-024-02481-z)
Supplement: Supplementary file 1 — Supplementary Material 1 [file 787_2024_2481_MOESM1_ESM.docx]

**Supplementary Materials**

**Table S1** Participant demographics

| Participant demographics | Mean (*SD*) | N (%) |
| --- | --- | --- |
| Adolescent age | 11.45 (0.77) |  |
| Adolescent race |  |  |
| White/Caucasian |  | 46 (71.9%) |
| Mixed Heritage |  | 9 (14.1%) |
| Asian |  | 5 (7.81%) |
| Aboriginal or Torres Strait Islander |  | 3 (4.69%) |
| Black |  | 1 (1.56%) |
| Mother race |  |  |
| White/Caucasian |  | 51 (79.7%) |
| Mixed Heritage |  | 3 (4.69%) |
| Asian |  | 8 (12.5%) |
| Aboriginal or Torres Strait Islander |  | 2 (3.12%) |
| Black |  | 0 (0%) |
| Mother highest education |  |  |
| Primary school or less |  | 0 (0%) |
| Partial high school |  | 1 (1.56%) |
| Completed high school |  | 1 (1.56%) |
| TAFE/Vocational training |  | 4 (6.25%) |
| Partial University degree |  | 3 (4.69%) |
| Completed three-year University degree |  | 20 (31.2%) |
| Completed Honours degree |  | 9 (14.1%) |
| Partial graduate school |  | 7 (10.9%) |
| Completed graduate school |  | 19 (29.7%) |
| Family yearly income in AUD |  |  |
| $100,000 or more |  | 47 (73.4%) |
| $60,000 - $99,999 |  | 10 (15.6%) |
| $40,000 - $59,999 |  | 5 (7.81%) |
| $20,000 - $39,999 |  | 1 (1.56%) |
| $19,999 or less |  | 1 (1.56%) |
| Family structure |  |  |
| Original family (both biological parents) |  | 50 (78.1%) |
| Step family |  | 2 (3.12%) |
| Sole parent family |  | 9 (14.1%) |
| Other |  | 3 (4.69%) |

**Table S2**

Valence and Arousal of Images used in the Cognitive Reappraisal Task

| Image set | Image type | Valence Mean (SD) | Arousal Mean (SD) |
| --- | --- | --- | --- |
| IAPS | Negative | 2.67 (1.63) | 5.60 (2.22) |
| OASIS | Negative | 2.38 (1.14) | 4.12 (1.81) |
| NAPS | Negative | 1.66 (0.98) | 2.24 (1.52) |
| IAPS | Neutral | 5.78 (1.52) | 4.10 (1.85) |
| OASIS | Neutral | 4.84 (1.05) | 3.26 (1.51) |
| NAPS | Neutral | 4.47 (1.00) | 3.16 (1.60) |

*Note.* IAPS = International Affective Picture System; NAPS = Nencki Affective Picture System; OASIS = Open Affective Standardized Image Set.

**Table S3** Results of Affect Label vs Shape Label Contrast

| Contrast | ROI or WB  analysis | Anatomical region | Family/parenting variable | MNI coordinates | | | Cluster size | *p*FWE |
| --- | --- | --- | --- | --- | --- | --- | --- | --- |
|  |  |  |  | x | y | z |  |  |
| Affect Label > Shape Label | WB | Middle frontal gyrus | Maternal emotion regulation | 42 | 8 | 62 | 77 | .002 |
|  | PFC ROI and WB | Superior frontal gyrus | Emotional climate of the family | 10 | 42 | 46 | 68 | .001 |
| Shape Label > Affect Label | PFC ROI | Superior frontal gyrus | Unsupportive emotion socialization | 10 | 42 | 46 | 45 | .008 |
|  | PFC ROI | Middle frontal gyrus | Unsupportive emotion socialization | 46 | 14 | 30 | 51 | .004 |
|  | PFC ROI | Inferior frontal gyrus | Unsupportive emotion socialization | 46 | 24 | 18 | 49 | .005 |
|  | WB | Temporal pole | Unsupportive emotion socialization | 58 | 10 | -30 | 97 | <.001 |

FWE = family wise error, PFC = prefrontal cortex, ROI = region of interest, WB = whole brain.

**Table S4** fMRI Results of Main Task Effects

| Contrast | Anatomical Region | MNI coordinates | | | Cluster size | *p*FWE |
| --- | --- | --- | --- | --- | --- | --- |
|  |  | x | y | z |  |  |
| Affect Label > Shape Label | Lateral occipital cortex | -38 | -86 | -14 | 10247 | <.001 |
|  | Right amygdala | 20 | -4 | -16 | 711 | <.001 |
|  | Left amygdala | -18 | -4 | -16 | 331 | <.001 |
|  | Middle frontal gyrus | 50 | 18 | 30 | 2263 | <.001 |
|  | Inferior frontal gyrus | -48 | 28 | 2 | 2644 | <.001 |
|  | Supramarginal gyrus | 50 | -36 | 6 | 452 | <.001 |
|  | Superior frontal gyrus | -10 | 10 | 56 | 625 | <.001 |
|  | Right thalamus | 18 | -30 | -0 | 77 | .002 |
|  | Superior temporal gyrus | -58 | -36 | 8 | 180 | <.001 |
|  | Frontal medial cortex | 6 | 48 | -18 | 55 | .019 |
|  | Postcentral gyrus | -50 | -30 | 66 | 50 | .032 |
| Shape Label > Affect Label | Lingual gyrus | -32 | -44 | -8 | 705 | <.001 |
|  |  | 32 | -40 | -10 | 691 | <.001 |
|  | Lateral occipital cortex | 42 | -76 | 36 | 10765 | <.001 |
|  |  | -58 | -62 | -8 | 619 | <.001 |
|  |  | 58 | -64 | -12 | 252 | <.001 |
|  | Precuneus cortex | -10 | -58 | 14 | 1695 | <.001 |
|  | Frontal pole | 32 | 38 | 42 | 1313 | <.001 |
|  | Precentral gyrus | -60 | -0 | 4 | 63 | .008 |
|  | Middle temporal gyrus | 66 | -20 | -8 | 133 | <.001 |
|  |  | 54 | -8 | -30 | 50 | .032 |
|  | Superior temporal gyrus | 62 | -0 | 4 | 147 | <.001 |
|  | Inferior temporal gyrus | -60 | -26 | -24 | 250 | <.001 |
|  | Superior parietal lobule | 20 | -48 | 74 | 54 | .021 |
| Affect Label > Observe | Lingual gyrus | 2 | -84 | 0 | 17124 | <.001 |
|  | Inferior frontal gyrus | -48 | 16 | 28 | 1790 | <.001 |
|  | Middle frontal gyrus | 50 | 24 | 30 | 852 | <.001 |
|  | Superior frontal gyrus | -8 | 10 | 54 | 393 | <.001 |
|  | Postcentral gyrus | -48 | -32 | 56 | 1067 | <.001 |
|  | Insular cortex | -32 | 24 | 2 | 260 | <.001 |
|  |  | 34 | 22 | 2 | 150 | <.001 |
|  | Cingulate gyrus | -4 | 4 | 30 | 92 | .001 |
|  | Paracingulate gyrus | 12 | 14 | 46 | 104 | <.001 |
|  | Frontal pole | -46 | 46 | -6 | 294 | <.001 |
|  |  | 32 | 52 | -8 | 65 | .012 |
|  | Left thalamus | -8 | -30 | -4 | 76 | .005 |
|  | Superior temporal gyrus | -58 | -36 | 6 | 65 | .012 |
| Observe >  Affect Label | Lateral occipital cortex | -38 | -80 | 42 | 3079 | <.001 |
|  | Precuneus cortex | -14 | -60 | 20 | 5854 | <.001 |
|  | Cingulate gyrus | 6 | 36 | -6 | 1523 | <.001 |
|  | Supramarginal gyrus | 66 | -44 | 38 | 2501 | <.001 |
|  | Middle frontal gyrus | -30 | 24 | 48 | 983 | <.001 |
|  |  | 30 | 32 | 46 | 546 | <.001 |
|  |  | 64 | -22 | -8 | 157 | <.001 |
|  | Planum polare | 44 | -12 | -8 | 174 | <.001 |
|  | Superior temporal gyrus | -46 | -12 | -0 | 184 | <.001 |
|  | Inferior temporal gyrus | 56 | -6 | -34 | 85 | .002 |
|  |  | -60 | -22 | -26 | 157 | <.001 |
|  | Middle temporal gyrus | -64 | -60 | -4 | 117 | <.001 |
|  | Percental gyrus | 64 | 4 | 12 | 92 | .001 |
|  | Superior frontal gyrus | 28 | 12 | 62 | 82 | .003 |
|  | Lingual gyrus | -30 | -42 | -10 | 68 | .009 |
|  | Superior parietal lobule | 26 | -40 | 62 | 63 | .015 |
|  | Juxtapositional lobule cortex | 4 | -4 | 68 | 64 | .013 |
| Look Negative > Look Neutral | Lateral occipital cortex | 50 | -78 | -4 | 10377 | <.001 |
|  | Frontal medial cortex | -4 | 44 | -18 | 418 | <.001 |
|  | Temporal pole | 38 | 22 | -28 | 384 | <.001 |
|  |  | -44 | 18 | -26 | 380 | <.001 |
|  | Middle temporal gyrus | 54 | -10 | -16 | 359 | <.001 |
|  |  | -64 | -6 | -16 | 222 | <.001 |
|  | Inferior temporal gyrus | -48 | -18 | -26 | 73 | .004 |
|  | Temporal fusiform cortex | 38 | -4 | -42 | 52 | .032 |
|  | Left amygdala | -20 | -6 | -14 | 135 | <.001 |
|  | Right amygdala | 20 | -4 | -14 | 128 | <.001 |
|  | Cingulate gyrus | 4 | -50 | 28 | 227 | <.001 |
| Look neutral > Look Negative | Superior parietal lobule | 26 | -40 | 68 | 572 | <.001 |
|  | Postcentral gyrus | -22 | -32 | 66 | 1259 | <.001 |
|  |  | 50 | -16 | 54 | 110 | <.001 |
|  | Central opercular cortex | -36 | 4 | 14 | 661 | <.001 |
|  |  | 46 | 4 | 4 | 1134 | <.001 |
|  | Supramarginal gyrus | -50 | -48 | 54 | 141 | <.001 |
|  | Frontal pole | -46 | 52 | 2 | 146 | <.001 |
|  |  | 42 | 54 | -14 | 71 | .005 |
| Reappraisal >  Look Negative | Middle frontal gyrus | -40 | 10 | 60 | 776 | <.001 |
|  | Inferior frontal gyrus | -56 | 32 | 8 | 1414 | <.001 |
|  | Superior motor cortex | -4 | 4 | 70 | 2437 | <.001 |
|  | Occipital pole | -22 | -94 | 4 | 257 | <.001 |
|  | Left caudate | -16 | 12 | 14 | 115 | <.001 |
|  | Middle temporal gyrus | -66 | -44 | 2 | 61 | .015 |
|  | Temporal pole | -52 | 18 | -30 | 176 | <.001 |
| Look Negative > Reappraisal | Supramarginal gyrus | -64 | -28 | 22 | 443 | <.001 |
|  |  | 60 | -32 | 36 | 199 | <.001 |
|  | Precentral gyrus | 12 | -32 | 46 | 65 | .010 |
|  | Postcentral gyrus | -26 | -44 | 74 | 143 | <.001 |
|  | Central opercular cortex | -48 | -2 | -0 | 332 | <.001 |
|  | Precuneus cortex | 18 | -68 | 34 | 73 | .005 |

*Note.* FWE = family wise error.

**Table S5** Significant associations between brain function during emotion regulation tasks and adolescent internalizing symptoms

| Contrast | ROI or WB  analysis | Anatomical region | MNI coordinates | | | Cluster size | *p*FWE |
| --- | --- | --- | --- | --- | --- | --- | --- |
|  |  |  | x | y | z |  |  |
| Shape Label > Affect Label | PFC ROI and WB | Middle frontal gyrus | 34 | 60 | -8 | 61 | .001 |
| Reappraisal > Look Negative | PFC ROI | Superior frontal gyrus | -4 | -4 | 76 | 33 | .041 |
|  | Amygdala ROI | Left amygdala | -18 | 2 | -18 | 11 | .013 |

FWE = family wise error, PFC = prefrontal cortex, ROI = region of interest.


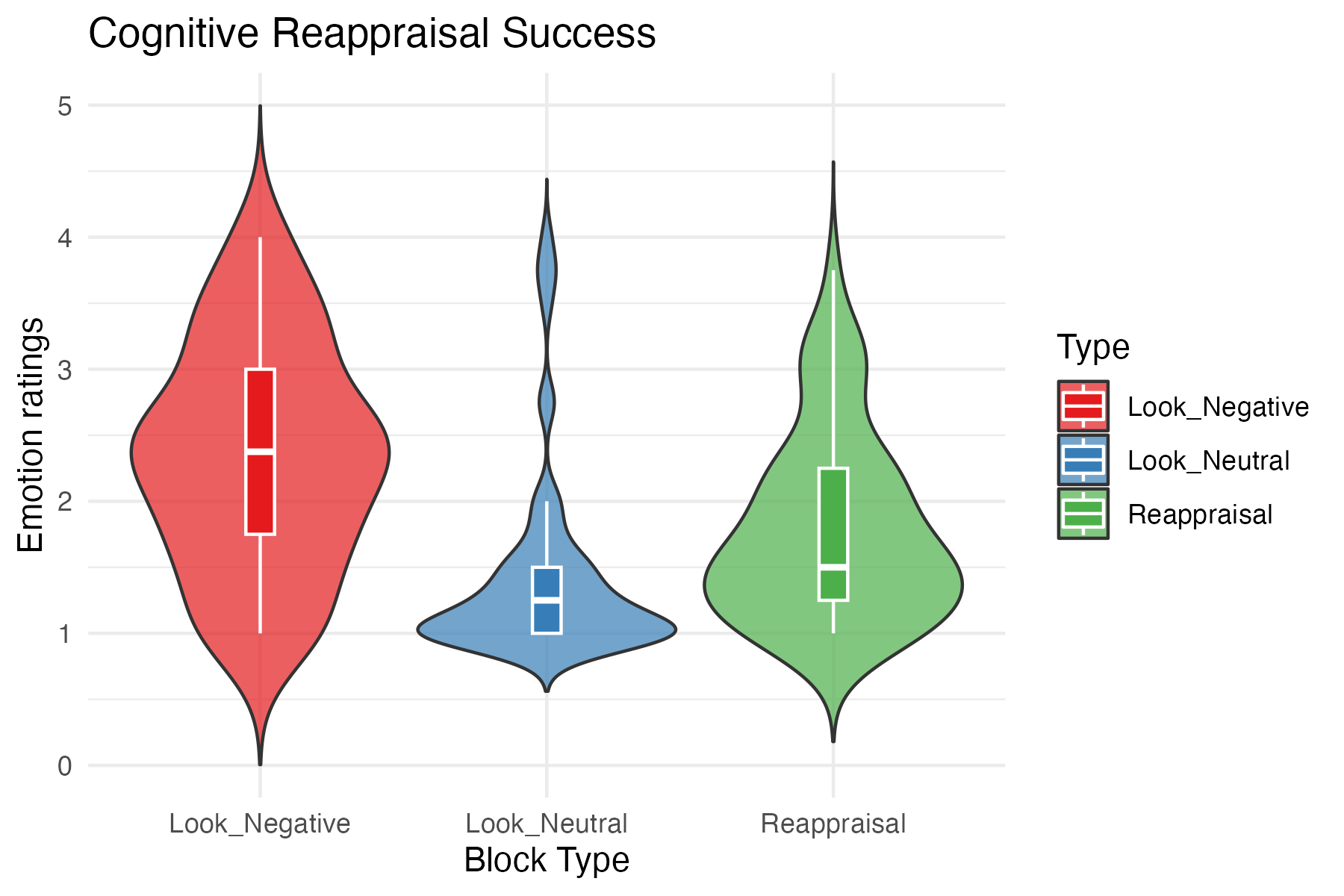


**Fig. S1**. Emotion ratings in the cognitive reappraisal task.
